# Do Hypoalbuminaemia Increase the Risk of Surgical Site Infection in Neck of Femur Fracture Patients: A Systematic Review and Meta-Analysis

**DOI:** 10.7759/cureus.61372

**Published:** 2024-05-30

**Authors:** Omar E Mostafa, Omar Al-Allaf, Muaaz Tahir, Fahad Hossain, John Blackwell

**Affiliations:** 1 General Surgery, Dudley Group National Health Service (NHS) Foundation Trust, Dudley, GBR; 2 Trauma and Orthopaedics, Walsall Healthcare National Health Service (NHS) Foundation Trust, Birmingham, GBR; 3 Trauma and Orthopaedics, Royal Orthopaedic Hospital, Birmingham, GBR

**Keywords:** nutrition, albumin, surgery, infection, trauma

## Abstract

Serum albumin plays an important role in physiological and inflammatory haemostasis, and low serum levels are linked with an increased incidence of surgical site infections (SSI). Although this has been demonstrated in the spine and elective arthroplasty settings, there is a paucity of evidence with regard to the effect of low serum albumin on rates of SSI following surgery for adult patients suffering from traumatic and acute hip fractures.

A systematic review was conducted using the PRISMA guidelines. Four databases were searched for randomised controlled trials (RCTs), cohort studies, and case-controlled studies. The risk of bias was assessed using the Newcastle-Ottawa Score (NOS). Data was collected and pooled using RevMan Web software. Results were reported as odds ratios (OR) with 95% confidence intervals (CI) and statistical significance of p <0.05. An inverse variance model was used in the meta-analysis.

Six retrospective studies (five cohorts and one case-control) with a total of 43,059 patients were included. 45.3% (n=19 496) had low serum albumin (<3.5 g/dL). Hypoalbuminemia was associated with a significantly higher risk of any form of SSI (OR 1.25, p=0.008) and deep SSI (OR 1.76, p=0.05). There was no statistical significance between hypoalbuminemia and the incidence of superficial SSI (OR 1.06, p=0.77). Organ-space SSI was associated with hypoalbuminemia, although one study reported this with poor statistical significance (OR 8.74, p<0.054).

Hypoalbuminemia increases the risk of most forms of surgical site infections, both superficial and deep. There is a weak conclusion to draw between the incidence of deep-space organ infections and low serum albumin.

## Introduction and background

Although the exact mechanisms are poorly understood, low serum albumin levels are associated with a high number of infectious diseases [1]. Among patients undergoing acute or elective orthopaedic surgery, a low serum albumin level (<3.5 g/dL) has been identified as a risk factor for surgical site infections (SSI) [2]. Hypoalbuminemia also increases the risk of other adverse outcomes such as mortality, infections, and cardiorespiratory complications [1].

Albumin is implicated in several physiological roles, including maintaining oncotic pressure, transportation of hormones and electrolytes, and antioxidant and antiplatelet effects [3,4]. Furthermore, albumin levels correlate to the level of nutrition, reflecting the underlying physiological reserve. Therefore, alterations in albumin levels have a fundamental impact on surgical practice and postoperative surgical care [5].

To date, however, there have been limited systematic reviews about the impact of hypoalbuminemia on the incidence of SSI in patients undergoing hip surgery following trauma. While this study acknowledges the work of Yuwen et al. and Li et al. [2,6], the current literature base does not delineate between hip fracture surgery and other types of orthopaedic surgeries, particularly primary arthroplasties of the hip, knee, or both.

The primary aim of this study is to analyse the effect of surgical site infections in patients with preoperative hypoalbuminemia undergoing surgery for acute, traumatic hip fractures.

## Review

Materials and methods

Design

This study was conducted in accordance with the Preferred Reporting Items for Systematic Reviews and Meta-Analyses (PRISMA) guidelines [7]. The inclusion criteria were developed and agreed upon by the authors of this systematic review. A literature search was conducted by two authors independently from conception to May 2023. No ethical approval was required for this study.

Inclusion Criteria

Only primary research studies were included in this review. Any abstracts, comments, letters, and reviews were excluded. The eligibility criteria included English-language studies of patients above the age of 18 who sustained a hip fracture requiring operative management (hemiarthroplasty, total hip arthroplasty (THA), extramedullary device/dynamic hip screw, intramedullary device) in the emergency setting. Hip fractures included the neck of the femur (intracapsular, extracapsular) or proximal femur. We excluded studies where patients underwent revision arthroplasty. The studies were required to report preoperative serum albumin levels and the incidence of SSI within 30 to 90 days postoperatively. Hypoalbuminemia is defined as serum albumin less than 3.5 g/dL, while normal serum albumin is defined as serum albumin of 3.5 g/dL or greater. Outcome: incidence of SSI.

Definition of Surgical Site Infections

As per the Centers for Disease Control and Prevention (CDC), a surgical site infection was defined as any infection of a surgical wound post-operatively within a 30-day to 90-day period. The patient may or may not have required medical or surgical intervention. Surgical site infections were divided into generic (not classified), superficial, deep, or organ space infections [8].

Search Methods

An electronic search was conducted in line with the best evidence approach, using two independent authors performing the searches. Keywords sought included hypoalbuminemia, serum albumin, malnutrition, hip fractures, neck of femur fractures, wound infections, and post-operative complications. Our search strategy is highlighted in Table 4 in the appendices. We searched four databases: OVID Medline, EMBASE, Cochrane, and CINAHL, until May 2023. An electronic search was conducted in conjunction with the local Evidence and Knowledge Services at Walsall Manor Hospital [9]. Google Scholar, PubMed, and Article-Citations were searched for relevant studies. ﻿

Data Extraction

Data collection and analysis were performed independently by two authors, and a third author was involved to resolve any discrepancies. Studies were extracted from relevant databases onto an Excel spreadsheet using Mendeley Reference Manager. Two authors independently eliminated duplicate studies, screened the title and abstract, and performed full-text article screening for inclusion. Data were extracted from relevant studies and divided into their respective measured outcomes based on the type of complication. Baseline characteristics of patients with hypoalbuminemia in each study were obtained for sample size, study type, reported outcome, mean age, ASA, BMI, smoking, steroid use, and wound classification (Table 1). Those were identified as particular risk factors by the National Institute for Health and Care Excellence (NICE) [8].﻿

**Table 1:** Baseline characteristics of patients with hypoalbuminemia in individual studies ORIF: Open reduction internal fixation, IM: Intra-medullary, SSI: Surgical site infections, BMI: Body mass index, ASA: American Society of Anesthesiologists, N/A: Not applicable

Author	Year	Type of Study	Type of Surgery	Outcome	Sample Size	% of Hypoalbuminemia	Age (mean, years)	BMI (mean, kg/m^2^)	ASA 3-4 (n%)	Smoking (%)	Wound Classification	Steroid Use (%)
Aldebeyan et al. [12]	2017	Retrospective Cohort	ORIF, Hemiarthroplasty, Total Hip Arthroplasty	Postoperative Complications, SSI	10117	46%	81.2	24.2	90.4%	50.8	N/A	7.6%
Pass et al. [13]	2022	Retrospective Cohort	IM Nail, Total Hip Arthroplasty, Hemiarthroplasty	Postoperative Complications, SSI	642	16.7%	85	22.8%	90.7%	N/A	N/A	N/A
Newman et al. [14]	2020	Retrospective Cohort	Total Hip Arthroplasty	Postoperative Complications, SSI	1667	34%	73	23.9	83.5%	21.8%	N/A	N/A
Ma et al. [15]	2019	Case Control	Arthroplasty	SSI	611	24%	N/A	N/A	N/A	N/A	N/A	N/A
Bohl et al. [16]	2017	Retrospective Cohort	ORIF, Hemiarthroplasty, Total Hip Arthroplasty, IM Nail	Postoperative Complications, SSI	17651	46%	N/A	N/A	N/A	48.7%	N/A	N/A
Chung et al. [17]	2018	Retrospective Cohort	ORIF, Hemiarthroplasty, Total Hip Arthroplasty, IM Nail	Postoperative Complications, SSI	12373	47%	80.1	22.4	92.2%	16.8%	N/A	8.9%

Risk of Bias Assessment

Included studies were assessed for risk of bias using the validated Newcastle Ottawa Score (NOS) [10]. Both case-control and cohort studies received a score out of 9 based on selectivity, comparability, and effect measures (Tables 2, 3).

**Table 2:** NOS of retrospective cohort studies One asterisk indicates a point

Author	Year	Selection				Comparability	Outcome			Total score	Total Quality
		Representativeness of the Exposed Cohort	Selection of the Non-Exposed Cohort	Ascertainment of Exposure	Demonstration That Outcome of Interest Was Not Present at Start of Study	Comparability of Cohorts on the Basis of the Design or Analysis	Assessment of Outcome	Was Follow-Up Long Enough for Outcomes to Occur	Adequacy of Follow Up of Cohorts		
Aldebeyan et al. [12]	2016	⋆	⋆	⋆	⋆	⋆	⋆	⋆	⋆	8	Good
Bohl et al. [16]	2017	⋆	⋆	⋆	⋆		⋆	⋆	⋆	7	Good
Chung et al. [17]	2018	⋆	⋆	⋆	⋆		⋆	⋆	⋆	7	Good
Newman et al. [14]	2020	⋆	⋆	⋆	⋆	⋆	⋆	⋆	⋆	8	Good
Pass et al. [13]	2022	⋆	⋆	⋆	⋆		⋆	⋆	⋆	7	Good

**Table 3:** NOS of case control studies One asterisk indicates a point

Author	Year	Selection				Comparability	Exposure			Total score	Total Quality
		Is the case definition adequate?	Representativeness of the cases	Selection of Controls	Definition of Controls	Comparability of cases and controls based on the design or analysis	Ascertainment of exposure	Same method of ascertainment for cases and controls	Non-Response rate		
Ma et al. [15]	2019		⋆	⋆	⋆		⋆	⋆		5	Fair

Statistical analysis

Missing data were accounted for by analysing only the available data, as suggested by the Cochrane Handbook for Systematic Reviews of Interventions. All statistical analyses were performed using the Online Review Manager Web (RevMan Web) [Computer program], version 4.12.0 (The Cochrane Collaboration, Copenhagen, 2022) [11]. Outcomes from controlled (comparative) studies were assessed by pooled odds ratios (OR) with 95% confidence intervals (CI) using an inverse-variance method. If OR was not available, it was calculated using a 2 x 2 table. Significant heterogeneity was set at I2 > 50%, p<0.1; a fixed-effect model was employed as heterogeneity was not significant. A p-value threshold of 0.05 was used for statistical significance. Sensitivity analysis was performed to assess the risk of bias in the confounding and reporting of outcomes by comparing the effects of fixed and random effects on the heterogeneity of the meta-analysis.

Results

**Figure 1:**
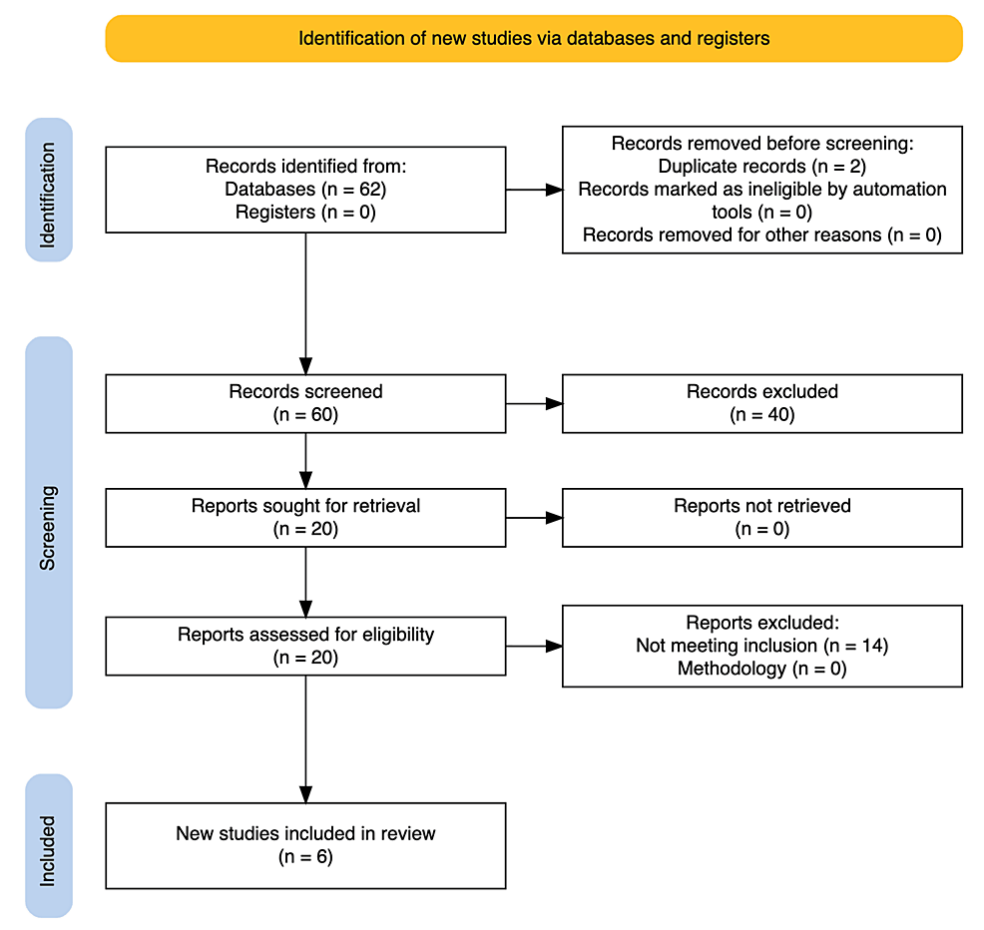
PRISMA diagram

This systematic review and meta-analysis included a total of 43,059 patients from six retrospective studies. Of which, 19 496 were in the hypoalbuminemia group, and 23 563 had normal serum albumin levels. With reference to the PRISMA flow diagram (Figure 1), a total of 20 studies were identified to undergo a full-text review after the removal of duplicates. Post-screening, a total of six studies were included in the qualitative and quantitative analysis of this systematic review. All included studies were observational studies: five retrospective cohort studies and one case-control study. Based on NOS criteria, the overall quality of the included studies was deemed good. All cohort studies had a low risk of bias in selection and outcome domains. The case-control study had a moderate risk of bias in selection and exposure domains. All studies had a high risk of bias in the comparability domain, except Aldebyan et al. [12] and Pass et al. [13], where a moderate risk of bias was observed. ﻿

Primary Outcome

**Figure 2:**
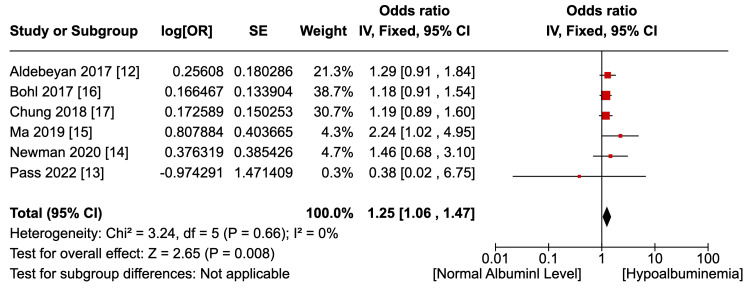
Overall SSI incidence (any type)

**Figure 3:**
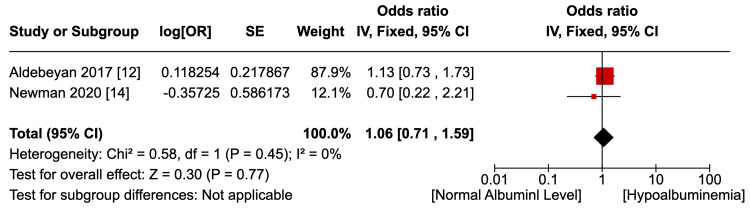
Incidence of superficial SSI

**Figure 4:**
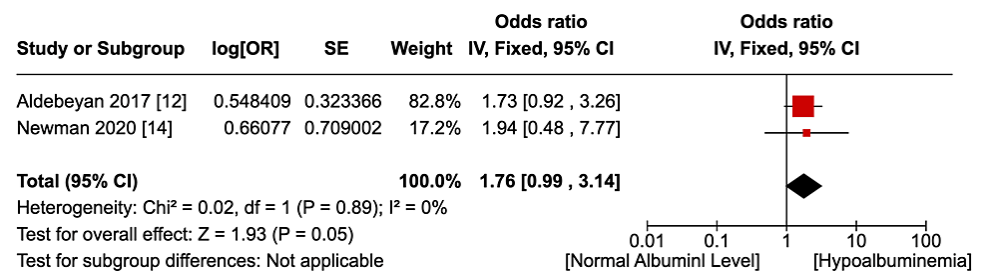
Incidence of deep SSI

Six studies [12-17] reported the outcome of an overall, uncategorised incidence of surgical site infections, as shown in Figure 2. Not accounting for confounding factors, hypoalbuminemia was associated with a statistically significant higher risk of any type of SSI (OR 1.25, p=0.008, 95% CI 1.06 to 1.47). Pooled data were homogenous (I2=0%, p=0.66) although not statistically significant. Two studies [12,14] reported the outcome of superficial SSI, as shown in Figure 3. There was no statistically significant difference in the risk of superficial SSI between the hypoalbuminemia and normal albumin groups (OR 1.06, p=0.77, 95% CI 0.71 - 1.59). Pooled data were homogenous (I2=0%, p=0.45) although lacking statistical significance. Two studies [12,14] reported the outcome of the incidence of deep SSI, as shown in Figure 4. Hypoalbuminemia was associated with an increased risk of deep SSI, which was statistically significant (OR 1.76, p=0.05, 95% 0.99 to 3.14). Pooled data were homogenous (I2=0%, p=0.89) but lacked statistical significance. Only one study reported an incidence of deep SSI; Newman et al. [14] found an increase in organ-space SSI in patients with hypoalbuminemia (OR 8.74, p<0.054), but the outcome was not statistically significant.

Discussion

The effects of serum albumin have long been identified as a predictor of outcomes in surgery [18]. This review aimed to analyse the impact of pre-operative serum albumin on the incidence of surgical site infections in adult patients undergoing surgery for a hip fracture.

This meta-analysis showed that hypoalbuminemia was associated with a higher risk of surgical site infections in this group of patients. Low serum albumin was also associated with an increased risk of deep and organ space-site infections.

These findings are consistent with previous studies in the trauma and orthopaedics literature. This study demonstrated an increase in any form of SSI by 1.25 times in the low albumin group, with no heterogeneity between studies. A systematic review and meta-analysis by Yuwen et al. in 2017, analysing the incidence of SSI in orthopaedic patients, demonstrated a significant correlation between low serum albumin levels and an increased risk of SSI in the spine, knee, and primary hip arthroplasties [2]. Albeit their reported heterogeneity, their analysis of 112,183 patients showed an increased rate of SSI by almost 2.5-fold. Similarly, in 2019, Li et al. analysed the prognostic role of serum albumin on the postoperative course after geriatric hip fractures, demonstrating a significant correlation between low serum albumin levels and increased total mortality and hospital deaths [6]. In their meta-analysis of 19 studies, hypoalbuminemia was 1.7 times more likely to cause postoperative complications, almost twice as likely. Their study concluded that routine use of admission albumin as a sole indicator of postoperative outcomes may be beneficial in fracture management [6]. This two-fold difference in reported studies can be attributed to the large number of pooled patients and studies analysed in their meta-analyses. Although the lack of reporting on surgical site infections limits the generalisability of the latter review, there is a demonstrated correlation between low albumin levels and rates of SSI.

Furthermore, this study sought to report correlations between low serum albumin and the incidence of organ-space infections. Only Newman et al. reported outcomes of organ space SSI, which were eight times more likely in patients with hypoalbuminemia, and increased major complications (i.e., pulmonary embolism) by 7% [14]. Due to the paucity of orthopaedic literature reporting the incidence of organ-space SSI, there is no clear consensus on the incidence of such a complication with hypoalbuminemia. Even outside of the orthopaedic literature, the reported effects of hypoalbuminemia have been contradictory. Tfaily et al. showed no correlation between low serum albumin and organ space infection in pancreatic surgery [19], while Hennessey et al. demonstrated that the increasing severity of hypoalbuminemia significantly increased the risk of organ space infections in patients undergoing gastrointestinal surgery [20]. This variation in outcomes is likely due to differences in methodology, study sites, and patient demographics. Although definitive conclusions cannot be drawn from these findings, it is an area that needs further exploration through controlled prospective studies.

Historically, biochemical markers such as albumin were considered to have a positive diagnostic value for malnutrition [21]. This is further supported by research on pre-operative nutritional optimisation through enteral or parenteral nutritional supplementation, which demonstrated a reduction in rates of infection post-abdominal surgery [22]. In patients with hip fractures, the role of perioperative optimisation using oral nutritional supplements remains an area of continued research. A systematic review by Chen et al. reported a significant reduction of infective complications by almost 50% in adult patients with hip fractures who received oral nutritional supplements [23]. Similarly, Oberstar et al. and Avenell et al. suggested a lower incidence of mortality and postoperative morbidity, including infections, in participants receiving oral multinutrient feeds [24,25]. However, future reviews need to evaluate the significance of nutritional supplementation on surgical wound infections, rates of return to the theater, and use of antibiotics.

Furthermore, much of the available literature on hypoalbuminemia synonymously uses the term 'malnutrition’. However, Soeters et al., The European Society for Clinical Nutrition and Metabolism (ESPEN), and the American Society for Parenteral and Enteral Nutrition (ASPEN) recommend that low serum albumin should not be diagnostic of malnutrition, as there is a correlation between inflammation and malnutrition rather than malnutrition and low serum albumin [1,21,26]. Similarly, another systematic review demonstrated that serum albumin and pre-albumin levels remained normal despite significant nutritional deprivation in patients with anorexia nervosa [27]. In patients with cancer, anthropometric measures were found to be superior to albumin in guiding oral nutritional supplementation [28]. This may be explained by the internal inflammatory response that is reproduced in both cancer and trauma situations. Therefore, this review suggests the use of alternative tools to assess malnutrition in the pre-operative phase to guide decision-making and perioperative optimisation; common methods such as subjective global assessment (SGA) and the perioperative nutrition screening tool (PONS) [26,29]. Future research should implement subgroup analysis to compare the utility and validity of various pre-operative nutritional assessment tools in predicting the incidence of surgical site infections.

The present study was subject to several potential methodological weaknesses. The principal limitation of this study is the inability to delineate other confounding factors for postoperative surgical site infections; SSI is influenced by multiple, simultaneous factors. Hence, we propose cautious application of the findings and propose subgroup analysis in future research. Despite this limitation, the high quality of the included studies, reported by NOS, supports the strong correlation between low albumin as a strong risk factor for SSI. Another source of uncertainty is the limited level of evidence; only retrospective cohort and case-control studies were analysed. However, there was no evidence of a difference in the pooled estimate with sensitivity analysis, particularly since all studies controlled for confounding appropriately. The reported outcomes are further strengthened by the homogeneity across all studies with a fixed effect model.

Furthermore, only one study reported results for organ-space SSI; with a wide confidence interval, careful interpretation of the applicability and generalisability of the results is therefore warranted. However, this result offers valuable insight into the possible negative implications of malnutrition on the postoperative course of patients undergoing lower limb surgery, suggesting an avenue for further research.

## Conclusions

Available literature has previously identified a negative correlation between serum albumin levels and postoperative recovery in other surgical specialties. The most prominent finding from this study is that low serum albumin increases the risk of surgical site infections. These findings have significant implications for understanding the perioperative optimisation of patients undergoing lower limb surgery. This information can also be used to develop targeted interventions aimed at patients with malnutrition in elective and emergency settings. Future studies should prospectively explore the effects of low serum albumin on long-term functional and subjective outcomes, such as rates of revision surgery, pain, and range of motion.

## Appendices

**Table 4:** Search strategy

Search Terms	Search Number
Hypoalbuminemia OR hypoalbuminemia OR serum albumin	#1
Fracture fixation OR hip fracture OR femoral neck	#2
#1 and #2	#3
Limit #3 to English Language Only	#4
